# Empyema necessitans: After recent thoracostomy in an immunocompromised patient

**DOI:** 10.1002/rcr2.1086

**Published:** 2023-01-26

**Authors:** Joana Almeida Borges, Daniela Madama

**Affiliations:** ^1^ Pulmonology Department Centro Hospitalar e Universitário de Coimbra Coimbra Portugal

**Keywords:** empyema, infection and inflammation, pleural diseases

## Abstract

This paper consists of a clinical image of an unexpected complication of a pleural space infection that dissects through the pleura into the soft tissues of the chest in an immunocompromised patient.

## Graphical abstract

Empyema necessitans is a rare complication of pleural space infections that dissects through the pleura into the soft tissues of the chest. This case describes an unexpected complication of an empyema treated in an immunocompromised patient.

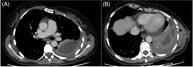

## CLINICAL IMAGE

A 51‐year‐old woman presents to the emergency department with pain and an area of swelling over the lateral left chest wall that has worsened in the previous week. She denied cough, dyspnea and fever.

She had a medical history of Waldenström macroglobulinemia (under bendamustine and rituximab combination) diagnosed by transthoracic lung biopsy that was complicated with a hemothorax. After discussion with thoracic surgery, she was managed with a left chest tube thoracostomy 4 months before.

**FIGURE 1:**
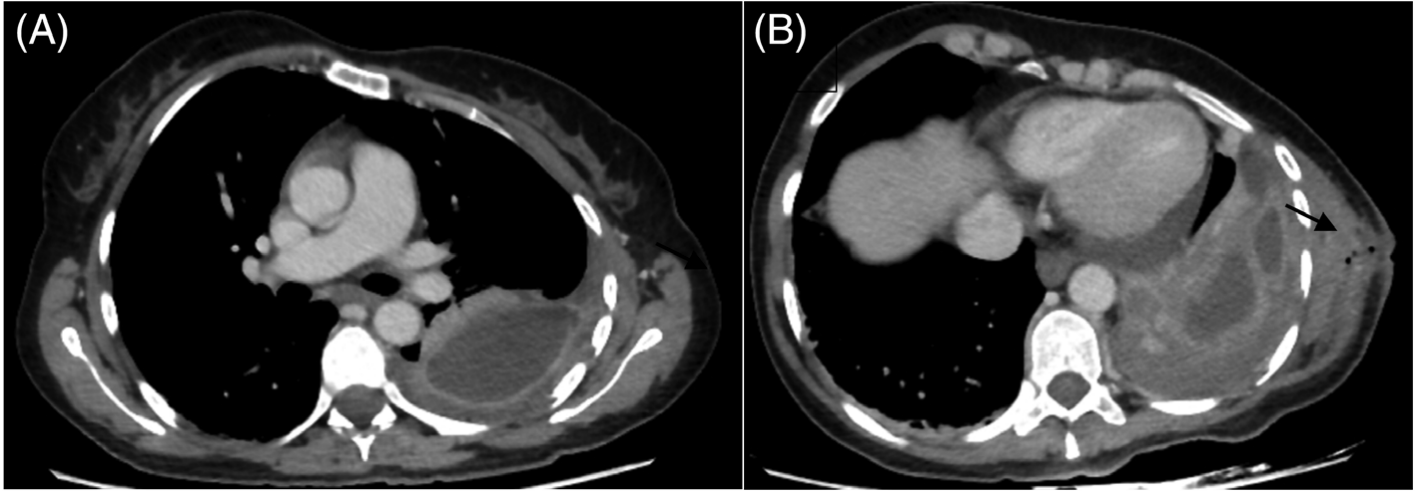
(A) Contrast thoracic CT axial cuts in mediastinum and lung windows showing the left pleural effusion with thickening of the pleura and split pleura sign. (B) Contrast thoracic CT axial cuts in mediastinum and lung windows showing the pleural fluid extended through a chest wall wound (wound opening—black arrow)

On emergency admission, the fluid collection on the left chest wall ruptured spontaneously and purulent material drained through the wound opening. Thoracic computed tomography (CT) scan shows a left pleural effusion (Figure 1A) with thickening of the pleura, split pleura sign and the pleural fluid extended through the chest wall (Figure 1B).

Thoracostomy tube placement was performed for 3 weeks, along with parenteral antibiotic treatment with ceftriaxone and clindamycin for 4 weeks. Cultures from the blood were negative and cultures from the purulent fluid isolated Streptococcus anginosus.

Empyema necessitans is a rare complication of pleural space infections that dissects through the pleura into the soft tissues of the chest.^1^ This case describes an unexpected complication of an empyema treated in an immunocompromised patient.

## AUTHOR CONTRIBUTIONS

Joana Almeida Borges conceived and developed the clinical image, carried out collection of data, wrote the first draft and collaborated in the final writing. Daniela Madama reviewed all the drafts critically and collaborated in the final writing. All the authors approved the final manuscript.

## FUNDING INFORMATION

There were no external funding sources for this study.

## CONFLICT OF INTEREST

The authors have no conflict of interest to declare regarding the present study.

## ETHICS STATEMENT

The authors declare that appropriate written informed consent was obtained for the publication of this manuscript and accompanying images.

## Data Availability

The data that support the findings of this study are available on request from the corresponding author. The data are not publicly available due to privacy or ethical restrictions.
